# 2,4,8,10-Tetra­oxa-3,9-dithia­spiro­[5.5]undecane 3,9-dioxide

**DOI:** 10.1107/S1600536808036891

**Published:** 2008-12-06

**Authors:** Zai-Ying Rao, Xin Xiao, Yun-Qiang Zhang, Sai-Feng Xue, Zhu Tao

**Affiliations:** a Key Laboratory of Macrocyclic and Supramolecular Chemistry of Guizhou Province, Guizhou University, Guiyang 550025, People’s Republic of China; b Institute of Applied Chemistry, Guizhou University, Guiyang 550025, People’s Republic of China

## Abstract

The asymmetric unit of the title compound, C_5_H_8_O_6_S_2_, consists of two spiro­[5.5]undecane mol­ecules. The nonplanar six-membered rings adopt chair conformations. In the crystal structure, weak inter­molecular C—H⋯O inter­actions, together with close O⋯S contacts in the range 3.308 (3)–3.315 (3) Å, stabilize the packing.

## Related literature

For background to the use of the title compound in the synthesis of pesticides, see: Jermy & Pandurangan (2005 ▶). For ring conformation puckering parameters, see: Cremer & Pople (1975 ▶). For bond-length data, see: Allen *et al.* (1987 ▶).
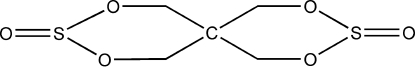


## Experimental

### 

#### Crystal data

C_5_H_8_O_6_S_2_
*M* *_r_* = 228.25
Orthorhombic, 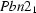

*a* = 6.0489 (5) Å
*b* = 12.8431 (11) Å
*c* = 21.5830 (18) Å
*V* = 1676.7 (2) Å^3^
*Z* = 8
Mo *K*α radiation
μ = 0.63 mm^−1^
*T* = 293 (2) K
0.25 × 0.23 × 0.19 mm

#### Data collection

Bruker SMART CCD area-detector diffractometer
Absorption correction: multi-scan (*SADABS*; Bruker, 2005 ▶) *T*_min_ = 0.858, *T*_max_ = 0.890
8878 measured reflections
1604 independent reflections
1531 reflections with *I* > 2σ(*I*)
*R*_int_ = 0.028

#### Refinement

*R*[*F* ^2^ > 2σ(*F* ^2^)] = 0.029
*wR*(*F* ^2^) = 0.078
*S* = 1.07
1604 reflections
235 parameters
1 restraint
H-atom parameters constrained
Δρ_max_ = 0.29 e Å^−3^
Δρ_min_ = −0.41 e Å^−3^
Absolute structure: Flack (1983 ▶), with 1497 Friedel pairs
Flack parameter: 0.09 (9)

### 

Data collection: *SMART* (Bruker, 2002 ▶); cell refinement: *SAINT* (Bruker, 2002 ▶); data reduction: *SAINT*; program(s) used to solve structure: *SHELXS97* (Sheldrick, 2008 ▶); program(s) used to refine structure: *SHELXL97* (Sheldrick, 2008 ▶); molecular graphics: *ORTEP-3 for Windows* (Farrugia, 1997 ▶); software used to prepare material for publication: *WinGX* (Farrugia, 1999 ▶).

## Supplementary Material

Crystal structure: contains datablocks global, I. DOI: 10.1107/S1600536808036891/at2669sup1.cif

Structure factors: contains datablocks I. DOI: 10.1107/S1600536808036891/at2669Isup2.hkl

Additional supplementary materials:  crystallographic information; 3D view; checkCIF report

## Figures and Tables

**Table 1:** Hydrogen-bond geometry (Å, °)

*D*—H⋯*A*	*D*—H	H⋯*A*	*D*⋯*A*	*D*—H⋯*A*
C2—H2*A*⋯O6^i^	0.97	2.53	3.343 (4)	141
C10—H10*A*⋯O9^i^	0.97	2.57	3.375 (4)	141
C3—H3*B*⋯O3^ii^	0.97	2.71	3.610 (4)	155
C9—H9*A*⋯O2^iii^	0.97	2.72	3.635 (4)	159

Symmetry codes: (i) 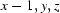; (ii) 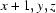; (iii) 
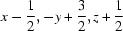
.

**Fig. 1.**
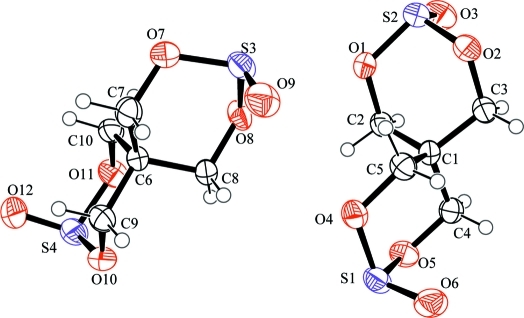
The molecular structure of (I) showing the atom-labelling scheme. Displacement ellipsoids are drawn at the 50% probability level.

## supplementary crystallographic information

### Comment

As part of our ongoing investigation into pentaerythritol compounds, we present an important intermediate in the synthesis of pesticides (Jermy & Pandurangan, 2005). The crystal structure determination of (I) has been carried out in order to elucidate the molecular conformation.

The crystal structure of the title compound, (I), consists of two spiro[5,5]undecane molecules (Fig. 1), in which the bond lengths are within normal ranges (Allen *et al.*, 1987). The four six-membered rings are not planar and adopt chair conformations, with a total puckering amplitude, Q_T_, of 0.607 (2) Å.

In the crystal structure, weak intermolecular C—H···O interactions, Table 1, together with close O5···S2 [d_O···S_ = 3.315 (3)] and O6···S3 [d_O···S_ = 3.308 (3)] contacts stabilize the packing.

### Experimental

A solution of thionyl chloride 30 ml (13.6 g 0.1 mol) in CH_2_Cl_2_ (30 ml) was added to a stirred solution of pentaerythritol (13.6 g 0.1 mol) in CH_2_Cl_2_ (50 ml) at room temperature for 24 h, and was then heated to reflux for 5 h. The resulting solution was evaporated to dryness under reduced pressure and the white product washed with warm water, the mixture filtered and the residue dissolved in 80 ml boiling distilled water then cooled. Single crystals of (I) were obtained after several days.

### Refinement

Water H atoms were located in a difference Fourier map and refined as riding in their as-found positions relative to O atoms with *U*_iso_(H) = 1.2*U*_eq_(O). All other H atoms were placed in calculated positions and refined as riding, with C—H = 0.93–0.97 Å, N—H = 0.86 Å, and *U*_iso_(H) = 1.2–1.5*U*_eq_(C,*N*).

### Crystal data

**Table:**

C_5_H_8_O_6_S_2_	*F*(000) = 944
*M**_r_* = 228.25	*D*_x_ = 1.808 Mg m^−^^3^
Orthorhombic, *P**b**n*2_1_	Mo *K*α radiation, λ = 0.71073 Å
Hall symbol: P 2c -2ab	Cell parameters from 1606 reflections
*a* = 6.0489 (5) Å	θ = 1.9–25.5°
*b* = 12.8431 (11) Å	µ = 0.63 mm^−^^1^
*c* = 21.5830 (18) Å	*T* = 293 K
*V* = 1676.7 (2) Å^3^	Prism, colourless
*Z* = 8	0.25 × 0.23 × 0.19 mm

### Data collection

**Table:**

Bruker SMART CCD area-detector diffractometer	1604 independent reflections
Radiation source: fine-focus sealed tube	1531 reflections with *I* > 2σ(*I*)
graphite	*R*_int_ = 0.028
φ and ω scans	θ_max_ = 25.5°, θ_min_ = 1.9°
Absorption correction: multi-scan (*SADABS*; Bruker, 2005)	*h* = −7→7
*T*_min_ = 0.858, *T*_max_ = 0.890	*k* = −15→15
8878 measured reflections	*l* = −25→25

### Refinement

**Table:**

Refinement on *F*^2^	Secondary atom site location: difference Fourier map
Least-squares matrix: full	Hydrogen site location: inferred from neighbouring sites
*R*[*F*^2^ > 2σ(*F*^2^)] = 0.029	H-atom parameters constrained
*wR*(*F*^2^) = 0.078	*w* = 1/[σ^2^(*F*_o_^2^) + (0.0528*P*)^2^ + 0.284*P*] where *P* = (*F*_o_^2^ + 2*F*_c_^2^)/3
*S* = 1.07	(Δ/σ)_max_ < 0.001
1604 reflections	Δρ_max_ = 0.29 e Å^−^^3^
235 parameters	Δρ_min_ = −0.41 e Å^−^^3^
1 restraint	Absolute structure: Flack (1983), with 1497 Friedel pairs
Primary atom site location: structure-invariant direct methods	Flack parameter: 0.09 (9)

### Special details

**Table:**

Geometry. All e.s.d.'s (except the e.s.d. in the dihedral angle between two l.s. planes) are estimated using the full covariance matrix. The cell e.s.d.'s are taken into account individually in the estimation of e.s.d.'s in distances, angles and torsion angles; correlations between e.s.d.'s in cell parameters are only used when they are defined by crystal symmetry. An approximate (isotropic) treatment of cell e.s.d.'s is used for estimating e.s.d.'s involving l.s. planes.
Refinement. Refinement of *F*^2^ against ALL reflections. The weighted *R*-factor *wR* and goodness of fit *S* are based on *F*^2^, conventional *R*-factors *R* are based on *F*, with *F* set to zero for negative *F*^2^. The threshold expression of *F*^2^ > σ(*F*^2^) is used only for calculating *R*-factors(gt) *etc*. and is not relevant to the choice of reflections for refinement. *R*-factors based on *F*^2^ are statistically about twice as large as those based on *F*, and *R*- factors based on ALL data will be even larger.

### Fractional atomic coordinates and isotropic or equivalent isotropic displacement parameters (Å^2^)

**Table:**

	*x*	*y*	*z*	*U*_iso_*/*U*_eq_	
C1	0.5760 (5)	0.8962 (2)	−0.21898 (13)	0.0267 (6)	
C2	0.3243 (5)	0.8908 (2)	−0.21098 (16)	0.0320 (7)	
H2A	0.2803	0.9325	−0.1756	0.038*	
H2B	0.2525	0.9189	−0.2475	0.038*	
C3	0.6435 (5)	0.8264 (3)	−0.27315 (14)	0.0336 (7)	
H3A	0.5837	0.8547	−0.3113	0.040*	
H3B	0.8033	0.8258	−0.2767	0.040*	
C4	0.6446 (5)	1.0070 (2)	−0.23563 (14)	0.0331 (7)	
H4A	0.8011	1.0084	−0.2453	0.040*	
H4B	0.5641	1.0296	−0.2721	0.040*	
C5	0.6937 (6)	0.8619 (3)	−0.15935 (16)	0.0332 (7)	
H5A	0.6417	0.7934	−0.1472	0.040*	
H5B	0.8516	0.8576	−0.1667	0.040*	
C6	0.0841 (5)	0.8707 (2)	0.04429 (13)	0.0282 (6)	
C7	0.1496 (6)	0.7587 (3)	0.06034 (15)	0.0380 (7)	
H7A	0.3062	0.7561	0.0699	0.046*	
H7B	0.0689	0.7363	0.0968	0.046*	
C8	0.2024 (6)	0.9046 (3)	−0.01531 (15)	0.0317 (7)	
H8A	0.1525	0.9734	−0.0273	0.038*	
H8B	0.3605	0.9077	−0.0081	0.038*	
C9	0.1562 (5)	0.9395 (3)	0.09843 (15)	0.0366 (7)	
H9A	0.0991	0.9107	0.1368	0.044*	
H9B	0.3163	0.9397	0.1010	0.044*	
C10	−0.1658 (5)	0.8766 (2)	0.03668 (16)	0.0344 (7)	
H10A	−0.2105	0.8358	0.0010	0.041*	
H10B	−0.2372	0.8477	0.0731	0.041*	
O1	0.2555 (4)	0.7835 (2)	−0.20175 (13)	0.0391 (6)	
O2	0.5638 (4)	0.72017 (16)	−0.26492 (13)	0.0393 (6)	
O3	0.1990 (5)	0.7482 (2)	−0.31274 (14)	0.0511 (7)	
O4	0.6503 (4)	0.93608 (18)	−0.10970 (10)	0.0384 (5)	
O5	0.6001 (4)	1.07751 (17)	−0.18509 (11)	0.0383 (5)	
O6	0.9676 (4)	1.0477 (2)	−0.13830 (14)	0.0483 (7)	
O7	0.1022 (4)	0.68874 (18)	0.00930 (12)	0.0414 (6)	
O8	0.1555 (4)	0.83071 (19)	−0.06494 (10)	0.0380 (5)	
O9	0.4707 (4)	0.7194 (2)	−0.03627 (15)	0.0532 (7)	
O10	0.0777 (4)	1.04518 (17)	0.09141 (13)	0.0430 (6)	
O11	−0.2349 (4)	0.9845 (2)	0.02836 (13)	0.0424 (6)	
O12	−0.2837 (5)	1.0115 (2)	0.13974 (14)	0.0618 (9)	
S1	0.73630 (15)	1.05361 (7)	−0.12165 (4)	0.0379 (2)	
S2	0.29956 (16)	0.70421 (6)	−0.25830 (4)	0.0384 (2)	
S3	0.24084 (16)	0.71310 (7)	−0.05390 (5)	0.0399 (2)	
S4	−0.18731 (16)	1.06085 (7)	0.08585 (5)	0.0446 (2)	

### Atomic displacement parameters (Å^2^)

**Table:**

	*U*^11^	*U*^22^	*U*^33^	*U*^12^	*U*^13^	*U*^23^
C1	0.0256 (15)	0.0307 (15)	0.0239 (14)	0.0010 (11)	0.0020 (11)	−0.0003 (11)
C2	0.0286 (17)	0.0333 (17)	0.0340 (17)	0.0035 (12)	0.0030 (13)	−0.0055 (13)
C3	0.0327 (16)	0.0354 (17)	0.0325 (17)	0.0003 (13)	0.0040 (12)	−0.0063 (12)
C4	0.0368 (17)	0.0334 (17)	0.0292 (14)	−0.0013 (13)	0.0011 (14)	0.0015 (12)
C5	0.0360 (17)	0.0324 (15)	0.0311 (16)	0.0027 (13)	−0.0013 (14)	0.0006 (14)
C6	0.0305 (16)	0.0303 (16)	0.0240 (14)	−0.0022 (12)	0.0025 (12)	−0.0006 (11)
C7	0.0466 (19)	0.0381 (18)	0.0295 (15)	−0.0005 (15)	0.0001 (15)	0.0056 (14)
C8	0.0369 (18)	0.0316 (16)	0.0265 (15)	−0.0014 (13)	0.0059 (12)	−0.0014 (12)
C9	0.0383 (18)	0.0416 (17)	0.0298 (16)	−0.0040 (14)	−0.0015 (14)	−0.0038 (14)
C10	0.0281 (17)	0.0380 (18)	0.0370 (17)	−0.0043 (13)	0.0031 (14)	−0.0091 (14)
O1	0.0367 (14)	0.0426 (14)	0.0380 (14)	−0.0083 (9)	0.0122 (9)	−0.0059 (11)
O2	0.0417 (14)	0.0312 (12)	0.0451 (13)	0.0043 (9)	0.0050 (11)	−0.0058 (10)
O3	0.0535 (16)	0.0548 (17)	0.0450 (15)	−0.0016 (13)	−0.0126 (12)	−0.0107 (12)
O4	0.0460 (14)	0.0413 (12)	0.0278 (11)	−0.0029 (11)	0.0013 (10)	−0.0014 (10)
O5	0.0423 (13)	0.0308 (12)	0.0418 (12)	0.0034 (9)	−0.0036 (11)	−0.0016 (10)
O6	0.0351 (13)	0.0505 (14)	0.0594 (17)	−0.0048 (11)	−0.0049 (12)	−0.0102 (12)
O7	0.0505 (15)	0.0306 (12)	0.0432 (13)	−0.0041 (10)	0.0035 (12)	−0.0017 (10)
O8	0.0489 (14)	0.0418 (13)	0.0232 (10)	0.0043 (10)	0.0018 (10)	−0.0021 (9)
O9	0.0399 (15)	0.0535 (16)	0.0663 (19)	0.0066 (12)	0.0044 (14)	−0.0147 (13)
O10	0.0471 (14)	0.0375 (12)	0.0445 (14)	−0.0105 (10)	0.0045 (13)	−0.0094 (10)
O11	0.0392 (15)	0.0431 (14)	0.0450 (15)	0.0095 (10)	−0.0050 (10)	−0.0082 (12)
O12	0.071 (2)	0.0582 (19)	0.056 (2)	−0.0121 (15)	0.0297 (15)	−0.0165 (15)
S1	0.0379 (5)	0.0403 (4)	0.0355 (5)	0.0005 (4)	−0.0027 (3)	−0.0106 (4)
S2	0.0431 (4)	0.0326 (4)	0.0397 (5)	−0.0040 (3)	0.0036 (4)	−0.0059 (4)
S3	0.0417 (5)	0.0397 (5)	0.0382 (5)	0.0028 (3)	0.0022 (3)	−0.0106 (4)
S4	0.0476 (5)	0.0371 (5)	0.0492 (6)	0.0018 (4)	0.0103 (5)	−0.0122 (4)

### Geometric parameters (Å, °)

**Table:**

C1—C4	1.526 (4)	C7—H7B	0.9700
C1—C3	1.528 (4)	C8—O8	1.458 (4)
C1—C2	1.534 (4)	C8—H8A	0.9700
C1—C5	1.535 (4)	C8—H8B	0.9700
C2—O1	1.454 (4)	C9—O10	1.446 (4)
C2—H2A	0.9700	C9—H9A	0.9700
C2—H2B	0.9700	C9—H9B	0.9700
C3—O2	1.458 (4)	C10—O11	1.458 (4)
C3—H3A	0.9700	C10—H10A	0.9700
C3—H3B	0.9700	C10—H10B	0.9700
C4—O5	1.443 (4)	O1—S2	1.612 (3)
C4—H4A	0.9700	O2—S2	1.618 (3)
C4—H4B	0.9700	O3—S2	1.439 (3)
C5—O4	1.458 (4)	O4—S1	1.617 (2)
C5—H5A	0.9700	O5—S1	1.627 (3)
C5—H5B	0.9700	O6—S1	1.446 (3)
C6—C10	1.522 (4)	O7—S3	1.632 (3)
C6—C9	1.528 (4)	O8—S3	1.614 (3)
C6—C7	1.532 (4)	O9—S3	1.444 (3)
C6—C8	1.535 (4)	O10—S4	1.620 (3)
C7—O7	1.450 (4)	O11—S4	1.608 (3)
C7—H7A	0.9700	O12—S4	1.447 (3)
			
C4—C1—C3	107.1 (2)	C6—C7—H7B	109.4
C4—C1—C2	109.8 (2)	H7A—C7—H7B	108.0
C3—C1—C2	108.9 (2)	O8—C8—C6	109.9 (2)
C4—C1—C5	109.8 (2)	O8—C8—H8A	109.7
C3—C1—C5	110.5 (2)	C6—C8—H8A	109.7
C2—C1—C5	110.7 (3)	O8—C8—H8B	109.7
O1—C2—C1	110.0 (2)	C6—C8—H8B	109.7
O1—C2—H2A	109.7	H8A—C8—H8B	108.2
C1—C2—H2A	109.7	O10—C9—C6	111.6 (3)
O1—C2—H2B	109.7	O10—C9—H9A	109.3
C1—C2—H2B	109.7	C6—C9—H9A	109.3
H2A—C2—H2B	108.2	O10—C9—H9B	109.3
O2—C3—C1	111.5 (2)	C6—C9—H9B	109.3
O2—C3—H3A	109.3	H9A—C9—H9B	108.0
C1—C3—H3A	109.3	O11—C10—C6	110.2 (2)
O2—C3—H3B	109.3	O11—C10—H10A	109.6
C1—C3—H3B	109.3	C6—C10—H10A	109.6
H3A—C3—H3B	108.0	O11—C10—H10B	109.6
O5—C4—C1	110.9 (2)	C6—C10—H10B	109.6
O5—C4—H4A	109.5	H10A—C10—H10B	108.1
C1—C4—H4A	109.5	C2—O1—S2	116.6 (2)
O5—C4—H4B	109.5	C3—O2—S2	117.12 (19)
C1—C4—H4B	109.5	C5—O4—S1	115.8 (2)
H4A—C4—H4B	108.0	C4—O5—S1	115.05 (18)
O4—C5—C1	110.2 (2)	C7—O7—S3	114.5 (2)
O4—C5—H5A	109.6	C8—O8—S3	116.0 (2)
C1—C5—H5A	109.6	C9—O10—S4	116.69 (19)
O4—C5—H5B	109.6	C10—O11—S4	115.7 (2)
C1—C5—H5B	109.6	O6—S1—O4	107.55 (15)
H5A—C5—H5B	108.1	O6—S1—O5	106.90 (16)
C10—C6—C9	109.7 (3)	O4—S1—O5	98.48 (12)
C10—C6—C7	109.1 (3)	O3—S2—O1	107.47 (16)
C9—C6—C7	107.2 (3)	O3—S2—O2	107.21 (16)
C10—C6—C8	111.0 (3)	O1—S2—O2	98.65 (12)
C9—C6—C8	110.1 (2)	O9—S3—O8	107.12 (14)
C7—C6—C8	109.6 (3)	O9—S3—O7	106.59 (18)
O7—C7—C6	111.0 (3)	O8—S3—O7	97.95 (13)
O7—C7—H7A	109.4	O12—S4—O11	106.32 (16)
C6—C7—H7A	109.4	O12—S4—O10	106.55 (18)
O7—C7—H7B	109.4	O11—S4—O10	99.11 (12)

### Hydrogen-bond geometry (Å, °)

**Table:**

*D*—H···*A*	*D*—H	H···*A*	*D*···*A*	*D*—H···*A*
C2—H2A···O6^i^	0.97	2.53	3.343 (4)	141
C10—H10A···O9^i^	0.97	2.57	3.375 (4)	141
C3—H3B···O3^ii^	0.97	2.71	3.610 (4)	155
C9—H9A···O2^iii^	0.97	2.72	3.635 (4)	159

Symmetry codes: (i) *x*−1, *y*, *z*; (ii) *x*+1, *y*, *z*; (iii) *x*−1/2, −*y*+3/2, *z*+1/2.
